# Joint effect of atrial fibrillation and obesity on mortality in critically ill patients

**DOI:** 10.1186/s13098-024-01407-8

**Published:** 2024-07-18

**Authors:** Hong-Da Zhang, Lei Ding, Li-Jie Mi, Ai-Kai Zhang, Yuan-Dong Liu, Fu-Hua Peng, Xin-Xin Yan, Yu-Jing Shen, Min Tang

**Affiliations:** State Key Laboratory of Cardiovascular Disease, Fuwai Hospital, National Center for Cardiovascular Diseases, Chinese Academy of Medical Sciences & Peking Union Medical College, 167 Beilishi Road, Xicheng District, Beijing, 100037 China

**Keywords:** Atrial fibrillation, Obesity, Mortality, Intensive care unit

## Abstract

**Background:**

The interplay between atrial fibrillation (AF) and obesity on mortality in critically ill patients warrants detailed exploration, given their individual impacts on patient prognosis. This study aimed to assess the associations between AF, obesity, and 1-year mortality in a critically ill population.

**Methods:**

Utilizing data from the Medical Information Mart for Intensive Care (MIMIC)-IV database, we conducted a retrospective analysis of adult patients admitted to the intensive care unit. The primary endpoint was 1-year mortality, analyzed through Cox regression with hazard ratio (HR) and Kaplan-Meier survival methods.

**Results:**

The study included 25,654 patients (median age 67.0 years, 40.6% female), with 39.0% having AF and 36.1% being obese. Multivariate COX regression analysis revealed that AF was associated with a 14.7% increase in the risk of 1-year mortality (*p* < 0.001), while obesity was linked to a 13.9% reduction in mortality risk (*p* < 0.001). The protective effect of obesity on mortality was similar in patients with (HR = 0.85) and without AF (HR = 0.86). AF led to a slightly higher risk of mortality in patients without obesity (HR = 1.16) compared to those with obesity (HR = 1.13). Kaplan-Meier survival curves highlighted that non-obese patients with AF had the lowest survival rate, whereas the highest survival was observed in obese patients without AF.

**Conclusions:**

AF significantly increased 1-year mortality risk in critically ill patients, whereas obesity was associated with a decreased mortality risk. The most adverse survival outcomes were identified in non-obese patients with AF.

**Supplementary Information:**

The online version contains supplementary material available at 10.1186/s13098-024-01407-8.

## Introduction

In the landscape of critical care medicine, both atrial fibrillation (AF) and obesity pose a significant challenge to outcomes. AF, the most common cardiac arrhythmia in clinical practice, is associated with increased morbidity and mortality, especially in the critically ill population [1–4]. It makes the clinical process more challenging by making patients more susceptible to thromboembolic events, worsening heart failure, and complicating the maintenance of hemodynamic stability [3, 4] Concurrently, the global rise in obesity has reached epidemic proportions, with its presence in the intensive care unit (ICU) setting raising concerns due to its potential impact on drug absorption and metabolism, increased mechanical ventilation times, and a predisposition to various comorbid conditions [5–7]. Nevertheless, the concept of the “obesity paradox” challenges traditional beliefs as it suggests that obesity may actually provide a survival benefit in specific groups of critically ill individuals, prompting a reassessment of its significance in this scenario [5–7].

The interplay between AF and obesity in critically ill patients is complex and not fully understood. Previous investigations have proposed that AF, a known risk factor for heightened mortality in the general and hospitalized populations, may have a less defined impact on the critically ill, particularly in relation to obesity. The presence of obesity could modulate the risk associated with AF through various mechanisms, including but not limited to metabolic reserve, inflammatory response, and differing cardiovascular dynamics [6, 7]. Therefore, exploring the combined impact of AF and obesity on mortality in the critically ill population is crucial for developing targeted interventions and improving patient outcomes.

This study aimed to bridge the gap in knowledge by utilizing a large cohort of critically ill patients to investigate the effects of AF and obesity on mortality. By examining the survival outcomes of patients with varying statuses of obesity and AF in an ICU setting, the present study provided insights into how these conditions interact and correlate with mortality risk. The results will serve as valuable guidance for medical decisions and resource distribution and encourage further investigation into individualized care for critically ill patients.

## Methods

### Data source and ethics

The Medical Information Mart for Intensive Care (MIMIC)-IV critical care database (version 2.2) was the source of patients for this study. The database included 431,231 hospitalizations, and 73,181 ICU stays between 2008 and 2019 [8–10]. Data for patients who were admitted to the emergency department or one of the ICU of Beth Israel Deaconess Medical Center, Boston, Massachusetts, were extracted from the respective hospital databases [8–10]. The first author, Dr. Zhang HD obtained access to the database and was responsible for data extraction (certification number 57,478,823). This database was exempted from our institutional review board approval. No patient-informed consent was necessary as the data had been completely de-identified.

### Cohort selection and data extraction

**Fig. 1:**
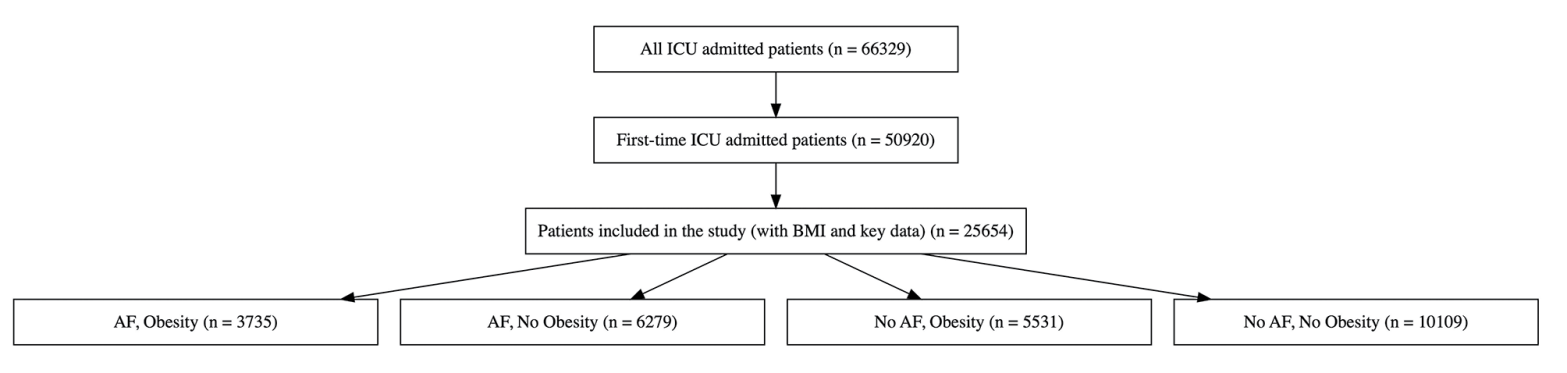
Flow chart for patient enrollment ICU = intensive care unit; AF = atrial fibrillation

The study encompassed critically ill patients admitted to the ICU, and the selection criteria are shown in Fig. 1. To avoid repetition, only the initial ICU admission was taken into account for patients with multiple admissions. The diagnosis information was obtained from the table ‘diagnoses_icd’ and ‘d_icd_diagnoses’ in the database, based on the International Classification of Diseases. We excluded participants with a body mass index (BMI) of less than 15 kg/m^2^ or over 60 kg/m^2^ because there might have been errors in registering their weight or height. Obesity was defined as BMI of greater than 30 kg/m^2^. The obesity group was further divided into three subcategories based on BMI levels: Class I Obesity: BMI 30-34.9 kg/m²; Class II Obesity: BMI 35-39.9 kg/m²; Class III Obesity: BMI ≥ 40 kg/m². Our study included patients with and without AF. For patients with AF, the following categories were included: Pre-existing AF, New-onset AF (patients who were diagnosed with AF for the first time during their ICU stay), Paroxysmal AF, and Persistent AF. Demographics, baseline vital signs, disease severity scores, comorbidities, baseline laboratory data, and treatment information were all extracted (Table 1**)**. The liver function data, blood lipid profile, and cardiac enzymes were not incorporated due to a significant amount of missing data exceeding 20%. The primary outcome was 1-year mortality. Patients with missing data for the above parameters were not included. Data extraction was performed using pgAdmin4 version 7.6.

**Table 1:** Demographic and clinical characteristics of patients stratified by obesity and AF status

Variables	Overall population (*n* = 25,654)		AF			No AF			*p*-value*
			Obesity (*n* = 3735)	No obesity (*n* = 6279)		Obesity (*n* = 5531)	No obesity (*n* = 10,109)		
Age, years	67.0 (55.7–77.5)		70.4 (62.6–77.6)	76.8 (67.5–84.2)		60.3 (50.1–69.6)	62.8 (50.7–74.0)		< 0.001
Age group, n (%)									< 0.001
< 65 years	11,530 (44.9%)		1201 (32.2%)	1230 (19.6%)		3504 (63.4%)	5595 (55.4%)		
65–74 years	6283 (24.5%)		1280 (34.3%)	1577 (25.1%)		1245 (22.5%)	2181 (21.6%)		
75–84 years	5222 (20.4%)		960 (25.7%)	2113 (33.7%)		622 (11.3%)	1527 (15.1%)		
≥85 years	2619 (10.2%)		294 (7.9%)	1359 (21.6%)		160 (2.89%)	806 (8.0%)		
Women, n (%)	10,420 (40.6%)		1516 (40.6%)	2382 (37.9%)		2435 (44.0%)	4087 (40.4%)		< 0.001
BMI, kg/m^2^	27.77 (24.2–32.3)		34.4 (31.9–38.3)	25.4 (22.8–27.6)		34.3 (31.8–38.3)	25.2 (22.5–27.4)		< 0.001
Ethnicity									< 0.001
White, n (%)	17,400 (67.8%)		2726 (73.0%)	4585 (73.0%)		3585 (64.8%)	6504 (64.3%)		
Black, n (%)	2189 (8.5%)		295 (7.9%)	358 (5.7%)		624 (11.3%)	912 (9.0%)		
Asian, n (%)	636 (2.5%)		25 (0.67%)	197 (3.1%)		48 (0.9%)	366 (3.6%)		
Hispanic/Latino, n (%)	838 (3.3%)		99 (2.7%)	130 (2.1%)		228 (4.1%)	381 (3.8%)		
Other/Unknown, n (%)	4591 (17.9%)		590 (15.8%)	1009 (16.1%)		1046 (18.9%)	1946 (19.3%)		
Marital status									< 0.001
Married, n (%)	12,079 (47.1%)		1868 (50.0%)	3221 (51.3%)		2528 (45.7%)	4462 (44.1%)		
Divorced, n (%)	1814 (7.1%)		292 (7.8%)	389 (6.2%)		430 (7.8%)	703 (7.0%)		
Single, n (%)	6567 (25.6%)		822 (22.0%)	1081 (17.2%)		1677 (30.3%)	2987 (29.6%)		
Widowed, n (%)	2905 (11.3%)		466 (12.5%)	1115 (17.8%)		397 (7.2%)	927 (9.2%)		
Other/Unknown, n (%)	2289 (8.9%)		287 (7.7%)	473 (7.5%)		499 (9.0%)	1030 (10.2%)		
Insurance									< 0.001
Medicare, n (%)	1773 (6.9%)		136 (3.6%)	205 (3.3%)		534 (9.7%)	898 (8.9%)		
Medicaid, n (%)	11,196 (43.6%)		1938 (51.9%)	3726 (59.3%)		1872 (33.9%)	3660 (36.2%)		
Other, n (%)	12,685 (49.5%)		1661 (44.5%)	2348 (37.4%)		3125 (56.5%)	5551 (54.9%)		
Admission type									< 0.001
Emergency/urgency, n (%)	18,407 (71.8%)		2587 (69.3%)	4303 (68.5%)		4036 (73.0%)	7481 (74.0%)		
Elective, n (%)	1296 (5.1%)		275 (7.4%)	514 (8.2%)		181 (3.3%)	326 (3.2%)		
Other, n (%)	5951 (23.2%)		873 (23.4%)	1462 (23.3%)		1314 (23.8%)	2302 (22.8%)		
Severity Scores									
SOFA	4.0 (2.0–7.0)		5.0 (3.0–8.0)	5.0 (3.0–8.0)		4.0 (2.0–7.0)	4.0 (2.0–6.0)		< 0.001
LODS	4.0 (2.0–6.0)		5.0 (3.0–7.0)	5.0 (3.0–7.0)		4.0 (2.0–6.0)	4.0 (2.0–6.0)		< 0.001
APS III	40.0 (30.0–55.0)		45.0 (33.0–60.0)	44.0 (33.0–59.0)		38.0 (28.0–53.0)	38.0 (28.0–52.0)		< 0.001
OASIS	32.0 (26.0–38.0)		33.0 (27.0–39.0)	34.0 (28.0–40.0)		31.0 (25.0–37.0)	31.0 (25.0–36.0)		< 0.001
SAPS II	36.0 (27.0–45.0)		39.0 (31.0–49.0)	40.0 (33.0–49.0)		33.0 (24.0–42.0)	33.0 (25.0–42.0)		< 0.001
SIRS	3.0 (2.0–3.0)		3.0 (2.0–3.0)	3.0 (2.0–3.0)		3.0 (2.0–3.0)	3.0 (2.0–3.0)		0.003
Vital signs									
Heart rate, bpm	85.8 (76.4–98.2)		87.1 (78.0-100.7)	85.3 (76.4–97.9)		86.8 (77.0-98.7)	85.2 (75.5–97.3)		< 0.001
Systolic blood pressure, mmHg	117.2 (108.2-129.6)		116.6 (108.0-128.4)	115.4 (106.9-126.5)		119.4 (110.2-132.8)	117.3 (108.0-129.8)		< 0.001
Diastolic blood pressure, mmHg	62.9 (56.4–71.0)		61.8 (55.7–69.6)	60.6 (54.6–68.1)		64.5 (57.8–73.2)	63.7 (57.1–71.8)		< 0.001
Mean blood pressure, mmHg	78.0 (72.1–86.1)		77.3 (71.7–84.6)	76.2 (70.9–83.3)		79.7 (73.2–88.4)	78.6 (72.5–86.7)		< 0.001
Respiratory rate, breaths per minute	19.2 (17.0-22.2)		20.0 (17.7–23.0)	19.5 (17.0-22.6)		19.4 (17.2–22.4)	18.7 (16.5–21.5)		< 0.001
Oxygen saturation, (%)	97.7 (96.3–98.9)		97.6 (96.3–98.6)	97.9 (96.6–99.0)		97.3 (95.9–98.5)	97.9 (96.5–99.0)		< 0.001
Comorbidities									
Hypertension, n (%)	11,523 (44.9%)		1739 (46.6%)	2844 (45.3%)		2730 (49.4%)	4210 (41.67%)		< 0.001
Coronary artery disease, n (%)	9929 (38.7%)		1898 (50.8%)	3169 (50.5%)		1850 (33.5%)	3012 (29.8%)		< 0.001
Myocardial infarction, n (%)	4970 (19.4%)		872 (23.4%)	1552 (24.7%)		964 (17.4%)	1582 (15.7%)		< 0.001
Cardiomyopathy, n (%)	928 (3.6%)		203 (5.4%)	346 (5.5%)		125 (2.3%)	254 (2.5%)		< 0.001
Heart failure, n (%)	6802 (26.5%)		1648 (44.1%)	2603 (41.5%)		1031 (18.6%)	1520 (15.0%)		< 0.001
Hyperlipidemia, n (%)	10,327 (40.3%)		1923 (51.5%)	2925 (46.6%)		2164 (39.1%)	3315 (32.8%)		< 0.001
Sleep apnea, n (%)	2483 (9.7%)		774 (20.7%)	344 (5.5%)		922 (16.7%)	443 (4.4%)		< 0.001
Peripheral vascular disease, n (%)	3327 (13.0%)		584 (15.6%)	1110 (17.7%)		536 (9.7%)	1097 (10.9%)		< 0.001
Cerebrovascular disease, n (%)	3713 (14.5%)		535 (14.3%)	1047 (16.7%)		679 (12.3%)	1452 (14.4%)		< 0.001
Dementia, n (%)	712 (2.8%)		75 (2.0%)	250 (4.0%)		63 (1.1%)	324 (3.2%)		< 0.001
Chronic pulmonary disease, n (%)	6341 (24.7%)		1184 (31.7%)	1774 (28.3%)		1380 (25.0%)	2003 (19.8%)		< 0.001
Rheumatic disease, n (%)	848 (3.3%)		140 (3.8%)	263 (4.2%)		138 (2.5%)	307 (3.0%)		< 0.001
Peptic ulcer, n (%)	698 (2.7%)		105 (2.8%)	163 (2.6%)		126 (2.3%)	304 (3.0%)		0.052
Liver disease, n (%)	3080 (12.0%)		414 (11.1%)	505 (8.0%)		867 (15.7%)	1294 (12.8%)		< 0.001
Diabetes, n (%)	7543 (29.4%)		1673 (44.8%)	1652 (26.3%)		2016 (36.5%)	2202 (21.8%)		< 0.001
Paraplegia, n (%)	1152 (4.5%)		143 (3.8%)	274 (4.4%)		223 (4.0%)	512 (5.1%)		0.002
Renal disease, n (%)	4957 (19.3%)		1110 (29.7%)	1627 (25.9%)		852 (15.4%)	1368 (13.5%)		< 0.001
Cancer, n (%)	3044 (11.9%)		354 (9.5%)	811 (12.9%)		580 (10.5%)	1299 (12.9%)		< 0.001
Aids, n (%)	151 (0.6%)		2 (0.1%)	14 (0.2%)		24 (0.4%)	111 (1.1%)		< 0.001
Laboratory data									
White blood cell, k/ul	10.9 (7.8–15.0)		11.7 (8.4–16.1)	10.9 (7.8–14.9)		11.4 (8.1–15.5)	10.5 (7.5–14.5)		< 0.001
Hemoglobin, g/dl	10.6 (9.0-12.2)		10.3 (8.9–12.0)	10.1 (8.7–11.7)		11.0 (9.3–12.6)	10.7 (9.2–12.3)		< 0.001
Hematocrit, %	31.9 (27.4–36.6)		31.4 (27.2–36.2)	30.8 (26.4–35.3)		33.0 (28.3–37.7)	32.2 (27.8–36.9)		< 0.001
Platelet, k/ul	182.0 (132.0-244.0)		178.0 (134.0-235.0)	167.0 (123.0-228.0)		192.0 (141.5-253.5)	186.0 (133.0-251.0)		< 0.001
Urea nitrogen, mg/dl	18.0 (13.0–28.0)		22.0 (15.0–36.0)	21.0 (15.0–33.0)		17.0 (12.0–26.0)	16.0 (11.0–24.0)		< 0.001
Serum creatinine, mg/dl	0.9 (0.7–1.3)		1.1 (0.8–1.7)	1.0 (0.7–1.5)		0.9 (0.7–1.3)	0.9 (0.7–1.2)		< 0.001
Glucose, mg/dl	0.8 (0.6-1.0)		0.8 (0.7–1.1)	0.8 (0.6-1.0)		0.7 (0.6-1.0)	0.7 (0.5–0.9)		< 0.001
Sodium, mEq/L	125.0 (104.0-157.0)		131.0 (109.0-168.0)	124.0 (103.0-152.0)		131.0 (107.0-166.0)	121.0 (102.0-152.0)		< 0.001
Calcium, mg/dL	139.0 (136.0-141.0)		139.0 (136.0-141.0)	139.0 (136.0-141.0)		139.0 (136.0-141.0)	139.0 (136.0-141.0)		0.578
Potassium, mEq/L	8.3 (7.9–8.8)		8.4 (7.9–8.8)	8.3 (7.9–8.7)		8.3 (7.9–8.8)	8.3 (7.8–8.8)		< 0.001
Chloride, mEq/L	4.1 (3.8–4.6)		4.3 (3.9–4.7)	4.2 (3.8–4.6)		4.2 (3.8–4.6)	4.1 (3.7–4.5)		< 0.001
Bicarbonate, mEq/L	105.0 (101.0-109.0)		105.0 (101.0-108.0)	106.0 (101.0-110.0)		105.0 (101.0-108.0)	105.0 (102.0-109.0)		< 0.001
Aniongap, mEq/L	23.0 (21.0–25.0)		23.0 (21.0–26.0)	23.0 (20.0–25.0)		23.0 (21.0–25.0)	23.0 (20.0–25.0)		< 0.001
Interventions									
Mechanical ventilation, n (%)	12,566 (49.0%)		1951 (52.2%)	3244 (51.7%)		2753 (49.8%)	4618 (45.7%)		< 0.001
CRRT, n (%)	1119 (4.4%)		291 (7.8%)	325 (5.2%)		244 (4.4%)	259 (2.6%)		< 0.001
Medications									
Antiplatelets, n (%)	14,376 (56.0%)		2632 (70.5%)	4452 (70.9%)		2693 (48.7%)	4599 (45.5%)		< 0.001
Lipid lowering drugs, n (%)	12,995 (50.7%)		2452 (65.7%)	3814 (60.7%)		2623 (47.4%)	4106 (40.6%)		< 0.001
Oral anticoagulants, n (%)	7133 (27.8%)		1951 (52.2%)	3034 (48.3%)		869 (15.7%)	1279 (12.7%)		< 0.001
Beta-blockers, n (%)	17,636 (68.8%)		3166 (84.8%)	5282 (84.1%)		3466 (62.7%)	5722 (56.6%)		< 0.001
DHP CCB, n (%)	5558 (21.7%)		876 (23.5%)	1384 (22.0%)		1270 (23.1%)	2028 (20.1%)		< 0.001
Non-DHP CCB, n (%)	2557 (10.0%)		799 (21.4%)	1254 (20.0%)		222 (4.0%)	282 (2.8%)		< 0.001
Class IC Antiarrhythmic drugs, n (%)	93 (0.4%)		40 (1.1%)	42 (0.7%)		2 (0.0%)	9 (0.1%)		< 0.001
Class III Antiarrhythmic drugs, n (%)	4411 (17.2%)		1501 (40.2%)	2469 (39.3%)		154 (2.8%)	287 (2.8%)		< 0.001
Digoxin, n (%)	1235 (4.8%)		392 (10.5%)	752 (12.0%)		24 (0.4%)	67 (0.7%)		< 0.001
ACEI, n (%)	6447 (25.1%)		1108 (29.7%)	1772 (28.2%)		1404 (25.4%)	2163 (21.4%)		< 0.001
ARB, n (%)	1969 (7.7%)		424 (11.4%)	546 (8.7%)		477 (8.6%)	522 (5.2%)		< 0.001
ARNI, n (%)	37 (0.1%)		8 (0.2%)	14 (0.2%)		7 (0.1%)	8 (0.1%)		0.070
MRA, n (%)	1318 (5.1%)		289 (7.7%)	296 (4.7%)		322 (5.8%)	411 (4.1%)		< 0.001
Loop diuretics, n (%)	15,680 (61.1%)		2985 (79.9%)	4659 (74.2%)		3264 (59.0%)	4772 (47.2%)		< 0.001
Thiazides/thiazide-like diuretics, n (%)	2605 (10.2%)		632 (16.9%)	684 (10.9%)		641 (11.6%)	648 (6.4%)		< 0.001
Oral glucose-lowering drugs, n (%)	2003 (7.8%)		446 (11.9%)	423 (6.7%)		595 (10.8%)	539 (5.3%)		< 0.001
Insulin, n (%)	17,534 (68.4%)		2962 (79.3%)	4514 (71.9%)		3853 (69.7%)	6205 (61.4%)		< 0.001
PPIs, n (%)	13,160 (51.3%)		2054 (55.0%)	3420 (54.5%)		2863 (51.8%)	4823 (47.7%)		< 0.001
Inotropes and vasopressors, n (%)	13,917 (54.3%)		2470 (66.1%)	4052 (64.5%)		2710 (49.0%)	4685 (46.3%)		< 0.001
Outcomes									
ICU mortality	1983 (7.7%)		317 (8.5%)	635 (10.1%)		351 (6.4%)	680 (6.7%)		< 0.001
In-Hospital mortality	2688 (10.5%)		423 (11.3%)	866 (13.8%)		455 (8.2%)	944 (9.3%)		< 0.001
6-month mortality	5289 (20.6%)		752 (20.1%)	1799 (28.7%)		832 (15.0%)	1906 (18.9%)		< 0.001
1-year mortality	6293 (24.5%)		925 (24.8%)	2094 (33.4%)		985 (17.8%)	2289 (22.6%)		< 0.001

AF: atrial fibrillation; APS III: Acute Physiologic Score III; LODS: Logistic Organ Dysfunction Score; OASIS: the Oxford Acute Severity of Illness Score; SAPS II: Simplified Acute Physiology Score II; SIRS: Systemic Inflammatory Response Syndrome; SOFA: Sequential Organ Failure Assessment; CRRT: continuous renal replacement therapy; DHB CCB: dihydropyridine calcium channel blocker; Non-DHB: non- dihydropyridine; ACEI: angiotensin-converting enzyme inhibitor; ARB: angiotensin-receptor blocker; ARNI: angiotensin receptor-neprilysin inhibitor; MRA: mineralocorticoid receptor antagonist; PPI: proton pump inhibitors; ICU: intensive care unit

*Comparisons among the four subgroups

### Statistical analysis

Continuous variables were presented as the median with the interquartile range (IQR), and categorical data were displayed as percentages or ratios. The Mann-Whitney U test was applied to compare two independent groups of continuous data, while the Kruskal-Wallis test was used for comparisons across multiple groups. For categorical data analysis, the chi-square test was employed. Kaplan-Meier curves, differentiated by obesity and AF status, were plotted, and log-rank p-values were calculated for survival analysis. Multivariate Cox regression analysis was used to explore the relationship between obesity, AF, and mortality, with hazard ratios (HRs) and 95% confidence intervals (CIs) calculated. All clinically significant covariates were included in the multivariate Cox regression models. Patients with incomplete data were excluded from the initial selection, so no data imputation was necessary. A p-value of less than 0.05 was deemed statistically significant. All data management and analysis were conducted using R software, version 4.3.2.

## Results

### Cohort characteristics

Figure 1 illustrates the flow chart of patient selection for our study. From an initial pool of 66,329 patients admitted to the ICU, we narrowed it down to 50,920 individuals who were first-time ICU admissions. Out of these, 25,654 patients were included in the final analysis, based on the availability of BMI and essential clinical data. The characteristics of the overall cohort and subgroups stratified by AF status and obesity are presented in Table 1. In the overall cohort, patients had a median (IQR) age of 67.0 (55.7, 77.5) years, and 10,420 (40.6%) were female. Among all patients, 10,014 (39.0%) of the patients had AF, and 9266 (36.1%) had obesity. Patients were then categorized into four groups: obese with AF (*n* = 3,735), non-obese with AF (*n* = 6,279), obese without AF (*n* = 5,531), and non-obese without AF (*n* = 10,109). For patients with AF, 2,722 had pre-existing AF, 7,292 had new-onset AF, 8,411 patients had paroxysmal AF, and 1,603 patients had persistent AF (Supplemental Table 1).

Age distribution across the groups showed significant differences (*p* < 0.001), with the obese without AF group being the youngest (median age 60.3 years) and the non-obese with AF group being the oldest (median age 76.8 years). In terms of gender, a higher proportion of women were observed in the obese without AF group (44.0%). Ethnicity also varied significantly across groups, with the highest percentage of white individuals in the non-obese with AF group (73.0%).

Vital signs and laboratory data, such as heart rate, blood pressure, and white blood cell count, varied significantly, with the obese groups generally presenting more severe derangements (*p* < 0.001). Comorbidities like hypertension, coronary artery disease, and diabetes were more prevalent in the obese groups, especially among those with AF (*p* < 0.001). Regarding severity scores, all measured scores demonstrated significant differences across the groups, indicating varying levels of illness severity (*p* < 0.001).

Medication usage was notably higher in the obese groups, particularly among those with AF, with significant differences in the use of antiplatelets, lipid-lowering drugs, and oral anticoagulants (*p* < 0.001). The need for interventions such as mechanical ventilation and continuous renal replacement therapy was also more common in these groups (*p* < 0.001).

### Outcomes and Kaplan-Meier survival analysis

The outcomes stratified by obesity and AF status are displayed in Supplemental Table 2. Mortality rates varied, with in-hospital mortality highest in the non-obese with AF group (13.8%) and lowest in the obese without AF group (8.2%). Besides, all mortality measures, including ICU, in-hospital, 6-month, and 1-year mortality, were significantly different across the groups, highlighting the impact of obesity and AF on outcomes in critically ill patients (*p* < 0.001).

**Fig. 2:**
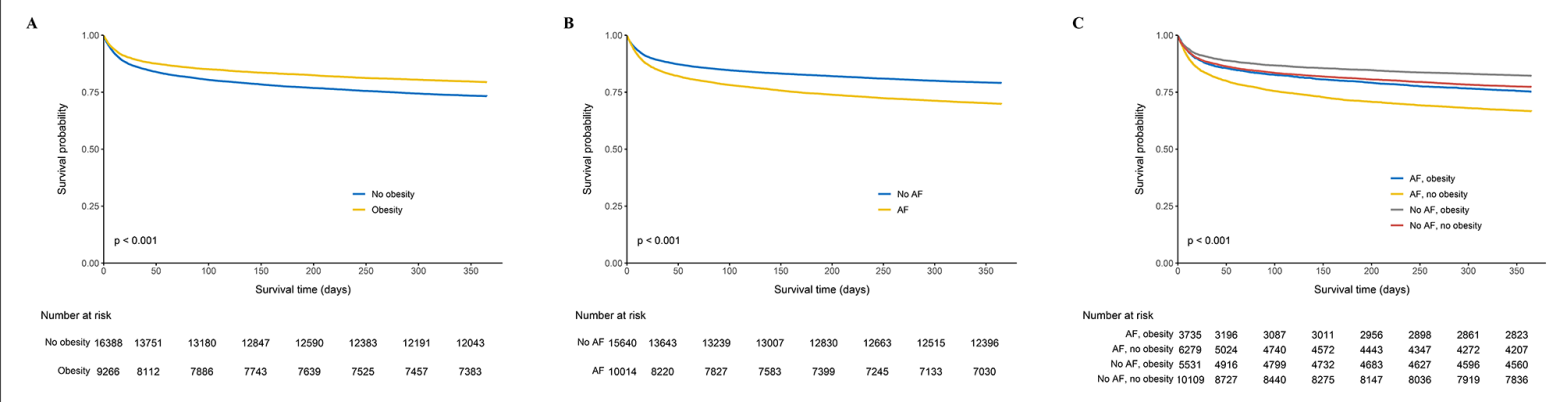
Kaplan-Meier curves of 1-year mortality stratified by obesity and AF status A: 1-year mortality in all patients stratified by obesity status; B: 1-year mortality in all patients stratified by AF status; C: 1-year mortality in all patients stratified by both obesity and AF status AF = atrial fibrillation

The Kaplan-Meier survival curves for patients stratified by obesity status (Fig. 2A) reveal a notable difference in survival probabilities over time (*p* < 0.001). Patients without obesity exhibited lower survival rates compared to those with obesity across the 1-year period. Initially, 16,388 non-obese patients and 9,266 obese patients were at risk, with the numbers declining more steeply for the non-obese group over time. Further analysis suggested that the mortality risk was similar across patients with Class I, II, and III obesity (Supplemental Figs.  1 and  2).

Survival analysis comparing patients with and without AF (Fig. 2B) shows that AF is associated with decreased survival over time (*p* < 0.001). The curves start with 15,640 patients without AF and 10,014 with AF, with the AF group experiencing a faster decline in survival probability over 1 year. Additional Kaplan-Meier survival curves (Supplemental Figs.  3– 5) demonstrate the survival outcomes for each AF category. Patients with new-onset AF exhibited higher 1-year mortality compared to those with pre-existing AF. Patients with persistent AF had worse survival outcomes compared to those with paroxysmal AF.

In Fig. 2C, the survival curves for four groups—AF with obesity, AF without obesity, no AF with obesity, and no AF without obesity—highlight the complex interplay between these conditions. The survival probability is highest in the no AF, obesity group, followed by those with no obesity and no AF. Patients with AF but no obesity, have the least favorable outcome (*p* < 0.001). The number at risk decreases over time, with the most significant drop in the AF, no obesity group.

### AF-related mortality in patients with and without obesity

Table 2 presents the analysis of 1-year mortality in relation to AF status among critically ill patients, stratified by obesity. Across the overall population, non-obese, and obese subgroups, patients with AF consistently exhibited higher mortality rates compared to those without AF. In the overall cohort, the unadjusted HR for 1-year mortality associated with AF was 1.516 (95% CI: 1.443–1.593, *p* < 0.001), indicating a significant increase in risk. When adjusting for age and gender in the basic model, the HR slightly decreased to 1.125 (95% CI: 1.068–1.186, *p* < 0.001). Further adjustments in the multivariate model, which included demographics, comorbidities, vital signs, laboratory parameters, disease severity scores, and treatments, yielded a HR of 1.147 (95% CI: 1.077–1.222, *p* < 0.001), maintaining the significant association between AF and increased mortality.

**Table 2:** AF and 1-year mortality in the overall population and subgroups with and without obesity

			Hazard Ratio (95% CI)										
	All patients						No Obesity				Obesity		
	No AF			AF	*p*-value		No AF	AF	*p*-value		No AF	AF	*p*-value
No. of events/person-days	3274/4,757,689			3019/2,782,022			2289/3,027,162	2094/1,681,247			985/1,730,528	925/1,100,775	
Unadjusted model	1 (Reference)			1.516 (1.443, 1.593)	< 0.001		1 (Reference)	1.570 (1.480, 1.666)	< 0.001		1 (Reference)	1.436 (1.313, 1.571)	< 0.001
Basic model	1 (Reference)			1.125 (1.068, 1.186)	< 0.001		1 (Reference)	1.141 (1.071, 1.216)	< 0.001		1 (Reference)	1.138 (1.035, 1.251)	0.007
Multivariate model	1 (Reference)			1.147 (1.077, 1.222)	< 0.001		1 (Reference)	1.155 (1.070, 1.247)	< 0.001		1 (Reference)	1.127 (1.005, 1.264)	0.041

Basic model: adjustment for age and gender

Multivariate model: basic model plus adjustment for other demographics, comorbidities, vital signs, laboratory parameters, disease severity scores, and treatment

CI: confidence interval; AF: atrial fibrillation

In the subgroup analysis, the pattern persisted. Among patients without obesity, the multivariate model showed that AF was associated with a higher risk of 1-year mortality (HR: 1.155, 95% CI: 1.070–1.247, *p* < 0.001). Similarly, in the obese subgroup, AF continued to be a significant predictor of 1-year mortality with a HR of 1.127 (95% CI: 1.005–1.264, *p* = 0.041) in the multivariate model.

### Obesity-related mortality in patients with and without AF

Table 3 analyzes the impact of obesity on 1-year mortality across the entire study population and within subgroups delineated by the presence or absence of AF. The result revealed that obesity was associated with a lower risk of 1-year mortality in the overall population, as well as within both the AF and no AF subgroups.

**Table 3:** Obesity and 1-year mortality in the overall population and subgroups with and without obesity

			Hazard Ratio (95% CI)										
	All patients						No AF				AF		
	No obesity			Obesity	*p*-value		No obesity	Obesity	*p*-value		No obesity	Obesity	*p*-value
No. of events/person-days	4383/4,708,409			1910/2,831,302			2289/3,027,162	985/1,730,528			2094/1,681,247	925/1,100,775	
Unadjusted model	1 (Reference)			0.743 (0.704, 0.784)	< 0.001		1 (Reference)	0.767 (0.712, 0.827)	< 0.001		1 (Reference)	0.701 (0.649, 0.757)	< 0.001
Basic model	1 (Reference)			0.821 (0.777, 0.866)	< 0.001		1 (Reference)	0.818 (0.759, 0.882)	< 0.001		1 (Reference)	0.817 (0.754, 0.884)	< 0.001
Multivariate model	1 (Reference)			0.861 (0.812, 0.912)	< 0.001		1 (Reference)	0.857 (0.791, 0.929)	< 0.001		1 (Reference)	0.848 (0.779, 0.923)	< 0.001

Basic model: adjustment for age and gender

Multivariate model: basic model plus adjustment for other demographics, comorbidities, vital signs, laboratory parameters, disease severity scores, and treatment

CI: confidence interval; AF: atrial fibrillation

For the overall patient cohort, the unadjusted model indicated an HR of 0.743 (95% CI: 0.704–0.784, *p* < 0.001) for obesity. After adjusting for age and gender in the basic model, the HR was 0.821 (95% CI: 0.777–0.866, *p* < 0.001). The multivariate model, which included a wider range of variables such as demographics, comorbidities, vital signs, laboratory parameters, disease severity scores, and treatments, provided an HR of 0.861 (95% CI: 0.812–0.912, *p* < 0.001), maintaining the significant association between obesity and lower mortality risk.

When stratified by AF status, the results remained consistent. In patients without AF, obesity was associated with a decreased 1-year mortality risk (multivariate model HR: 0.857, 95% CI: 0.791–0.929, *p* < 0.001). Similarly, among those with AF, obesity was linked to a lower risk of 1-year mortality (multivariate model HR: 0.848, 95% CI: 0.779–0.923, *p* < 0.001).

## Discussion

AF and obesity have both been found to have separate associations with outcomes among critically ill individuals in the field of critical care, underscoring the necessity for a comprehensive evaluation of their joint effects on mortality. By utilizing data from the MIMIC-IV database, we sought to better understand the interplay between AF and obesity and their overall effect on the survival of critically ill individuals. With a wide range of patients included, this comprehensive database provides a strong basis for analyzing clinical outcomes among different subpopulations. The results of our study demonstrate a subtle interplay, showing that AF increases the likelihood of mortality, while obesity is associated with a decreased mortality risk in this particular population.

Critical care patients with AF are more likely to experience unfavorable outcomes, contributing to the already high morbidity and mortality rates in this population [11, 12]. Its association with increased mortality in our analysis aligns with previous research, which attributes this heightened risk to the arrhythmia’s potential to precipitate hemodynamic instability, exacerbate underlying heart failure, and increase the propensity for thromboembolic events. The mortality risk linked to AF may be affected by its interaction with other prevalent comorbidities in critically ill individuals, including sepsis, heart failure, and chronic kidney disease [1–3, 11, 12]. With its inherently stressful conditions and the physical toll it takes on patients, the critical care setting can exacerbate the effects of AF, leading to a domino effect of complications that may impact survival. Given these complexities, the management of AF in the ICU requires a different approach that goes beyond rhythm control to include a holistic assessment of the patient’s overall health status, underlying comorbidities, and potential triggers of AF. Individualized therapy, combined with a thorough evaluation of the potential risks and benefits of various management techniques, is essential in achieving positive outcomes [4, 11, 12]. This includes not only pharmacological interventions, such as rate control and anticoagulation but also non-pharmacological approaches like electrical cardioversion or catheter ablation in selected cases. Our findings, which point to a 14.7% uptick in one-year mortality associated with AF, highlight the arrhythmia’s considerable impact and the crucial role of vigilant monitoring and proactive management in the ICU.

We included all critically ill patients with and without AF in our study. The reasons for admission encompassed a wide range of both cardiac and non-cardiac conditions in the MIMIC-IV database. Specifically, AF was present as both a primary reason for admission and as a comorbidity secondary to other underlying conditions. The detailed reasons for admission included: (1) AF: Some patients were admitted primarily due to AF, either newly diagnosed or pre-existing; (2) Cardiac Diseases: This included admissions for other cardiac conditions such as heart failure, myocardial infarction, and other arrhythmias where AF could be a secondary condition. (3) Non-Cardiac Diseases: A significant portion of the cohort was admitted for non-cardiac conditions such as sepsis, respiratory failure, renal failure, and other critical illnesses. In these cases, AF was often present as a comorbidity. By including this broad spectrum of admission reasons, our study aimed to capture the diverse clinical scenarios in which AF can occur, providing a comprehensive analysis of its impact on mortality among critically ill patients. Furthermore, we included all comorbidities as covariates in our multivariate analyses, allowing us to adjust for its potential confounding effects on mortality outcomes.

On the other hand, our findings suggest that obesity was associated with lower mortality, further fueling the debate surrounding the “obesity paradox.” The concept of the “obesity paradox” refers to the observation that, contrary to general expectations, obesity appears to confer a protective effect in certain populations, including critically ill patients [13, 14]. The findings of this study support previous research indicating that obesity may have a protective effect on mortality, as higher body mass index values have been linked to improved outcomes in critical conditions [13, 14]. There may be multiple components to the protective mechanism of obesity in critical illness, which could include physiological, metabolic, and immunological factors that provide resilience during acute medical emergencies [6, 7, 13, 14]. For instance, individuals who are obese may have a greater metabolic reserve, providing them with a vital advantage in managing catabolic stress during a critical illness [15]. In addition, the adipose tissue presents in those who are obese may serve as a defense mechanism by controlling pro-inflammatory cytokines and storing additional nutrients that can be released during times of acute illness [16]. Furthermore, the link between obesity and survival in critically ill patients could be affected by healthcare approaches, including nutritional aid and dosage of medications [17, 18]. Managing obese patients in the ICU often involves adapting medication dosages and providing personalized nutritional support, which could unintentionally result in more attentive and individualized care, potentially positively affecting outcomes [17, 18]. By juxtaposing the potential benefits of obesity with its established risks, the complexity of its role in the ICU becomes apparent, emphasizing the crucial need for a more thorough understanding of its biological and clinical implications. Future research should focus on elucidating the precise mechanisms underlying the obesity paradox and determining how these insights can be leveraged to improve outcomes for all critically ill patients, regardless of their BMI.

Even though the obesity paradox may imply some positive aspects of being overweight, it is crucial to be cautious as obesity is associated with significant health risks in the long run, such as heart disease, diabetes, and high blood pressure [19, 20]. Despite the temporary improvement in survival rates seen in the ICU, it is crucial to prioritize addressing obesity as a significant public health concern. Future research must focus on uncovering the intricate biological and clinical relationships that explain the obesity paradox, pinpointing the specific factors of obesity that contribute to this beneficial outcome and determining if they can be utilized for therapeutic purposes. When dealing with obese patients in the ICU, clinicians should keep in mind the distinctive physiological and pharmacological challenges they may encounter. This includes customizing treatment plans to optimize outcomes, as well as planning for long-term health and weight maintenance.

Examining the negative consequences of AF and the positive influence of obesity in critically sick patients sheds light on the intricate and counterintuitive characteristics of these frequently encountered clinical ailments [4, 13, 21]. The coexistence of these two opposing forces not only disrupts accepted medical beliefs but also emphasizes the need for a careful and detailed approach to treating patients in the ICU. It is crucial to comprehend and tackle the different ways in which AF and obesity impact patient journeys in order to achieve the best results for this susceptible group. In our study, the Kaplan-Meir curves (Fig. 2) collectively illustrate that while obesity appears to confer a survival benefit in the critically ill, AF significantly worsens survival, with the worst survival observed in non-obese patients with AF. However, in the multivariate COX survival analyses, our results suggested that for patients with obesity, AF did not pose a higher mortality risk compared to those without obesity (Table 2). Similarly, in patients with AF, obesity did not result in a reduced mortality risk when compared to those without AF (Table 3). Collectively, both AF and obesity were not found to have a significant impact on mortality risk in relation to each other. The interdependent nature of AF and obesity in critical care highlights the need for a thorough examination to uncover their joint influence on patient mortality.

*It is worth mentioning that a significant portion of patients had missing BMI or key data*,* primarily due to the following reasons: (1) A total of 23*,*413 patients were missing the height variable. This is a critical component for calculating BMI*,* and its absence was due to it not being consistently recorded in the MIMIC-IV database. (2) Other key laboratory variables were missing for a subset of patients because these measurements were either not ordered or not recorded during their ICU stay. This includes data such as liver function tests*,* blood lipid profiles*,* and cardiac enzyme levels*,* which were excluded from our analysis due to missing values exceeding 20%. To mitigate the impact of this missing data on our analysis*,* we took the following steps: (1) We excluded patients with missing BMI or critical laboratory variables to ensure the integrity and accuracy of our statistical models. This approach*,* while reducing our sample size*,* was necessary to avoid bias introduced by imputation or assumptions about the missing data. (2) By focusing on patients with complete data for the variables of interest*,* we ensured that our findings were based on reliable and comprehensive information. We recognize that missing data is a common challenge in large retrospective studies using electronic health records. However*,* we believe that our rigorous data handling and analysis methods have minimized the potential bias and ensured the robustness of our conclusions.*

Our study reveals a notable bias in the age distribution and survival rates between non-obese and obese patients, as indicated in Table 1. Non-obese patients tend to be older and exhibit different survival outcomes compared to their obese counterparts. These biases can have significant implications for the interpretation of our findings and warrant careful consideration. Non-obese patients in our cohort were generally older, which is consistent with existing literature indicating that older adults are more likely to have lower BMI [22, 23]. The advanced age of non-obese patients may contribute to higher mortality rates, independent of their obesity status. To account for this, we included age as a covariate in our multivariate analyses, allowing us to adjust for its potential confounding effects on mortality outcomes. While our statistical adjustments aim to mitigate these biases, it is important to acknowledge that residual confounding may still exist. The observed differences in age and survival rates underscore the complexity of interpreting the obesity paradox in critically ill patients. These biases highlight the need for further research to explore the nuanced interactions between age, obesity, and critical illness outcomes.

Our research methodology involved a thorough analysis of mortality rates in critically ill patients with AF and obesity, using the extensive and detailed MIMIC-IV database. The extensive dataset, encompassing a wide range of clinical variables and outcomes, provided a robust foundation for our analysis [8, 9]. The broad scope of data provided allowed for an in-depth analysis of intricate associations and the extraction of subtle observations on the interplay between AF and obesity in a critical care context. The strength of our study lies in the meticulous cohort selection and data extraction processes, ensuring a representative and relevant sample of the ICU population. By focusing exclusively on first-time ICU admissions, we minimized the potential confounding effects of repeated admissions, thereby enhancing the clarity and specificity of our findings. The utilization of standardized diagnostic codes and the extraction of detailed demographic, clinical, and treatment-related data allowed for a thorough and precise characterization of the study population.

### Limitations

Our study, while providing significant insights into the impact of AF and obesity on mortality in critically ill patients, has several limitations that merit consideration. First, the inherent nature of retrospective analyses limits the ability to establish causality. The findings are based on observational data from the MIMIC-IV database, which, despite its comprehensive nature, may be subject to biases related to data entry, coding inaccuracies, and the retrospective collection of clinical information. Second, the exclusion of patients with significant missing data, while necessary to maintain the integrity of the statistical analysis, may introduce selection bias. This exclusion could potentially omit a subset of patients who have different characteristics or worse outcomes, thus affecting the generalizability of our findings. The reliance on electronic health records for data collection also means that nuances in patient history and clinical presentation might not be fully captured, which could influence the study’s outcomes and interpretations. Third, our study’s findings are based on a single-center database, which may limit the applicability of the results to other settings or populations. Differences in healthcare systems, patient demographics, and clinical practices could result in variability in outcomes that might not be reflected in our analysis. Therefore, the external validity of our conclusions might be restricted, necessitating cautious interpretation when applying these findings to broader, more diverse populations. Additionally, while we adjusted for numerous confounders in our multivariate analyses, there remains the possibility of residual confounding due to unmeasured or inadequately measured variables. Factors such as socioeconomic status, lifestyle behaviors, and genetic predispositions, which are not comprehensively captured in the MIMIC-IV database, could influence the relationship between AF, obesity, and mortality in critically ill patients. Moreover, the study focused on the association of AF and obesity with mortality, potentially overlooking other important outcomes such as quality of life, functional status post-ICU discharge, and long-term morbidity. These outcomes are crucial for understanding the full spectrum of implications associated with these conditions in critically ill patients and warrant further investigation. Lastly, our findings demonstrate associations rather than causal relationships between AF, obesity, and mortality. While obesity was associated with decreased mortality risk, it cannot be concluded from this study that obesity directly reduces mortality. Future research should aim to replicate and extend these findings through prospective studies and randomized controlled trials, which can offer more controlled environments to explore the causal relationships and mechanisms underlying the observed associations.

## Conclusions

Our findings reveal a complex interplay where AF significantly increases mortality risk, while obesity was associated with a lower risk of mortality. Specifically, AF was associated with a 14.7% increase in one-year mortality risk, whereas obesity was linked to a 13.9% reduction in the same, underscoring the differential influence of these conditions in the ICU setting. Overall, the most adverse survival outcomes were identified in non-obese patients with AF. However, both AF and obesity were not found to have a significant impact on mortality risk in relation to each other. Prospective studies are needed to confirm these findings and explore the implications for clinical practice, including the development of guidelines for managing AF and assessing the role of obesity in critical care.

### Electronic supplementary material

Below is the link to the electronic supplementary material.

Supplementary Material 1

## Data Availability

No datasets were generated or analysed during the current study.

## Abbreviations

ACEI: Angiotensin-converting enzyme inhibitor
AF: Atrial fibrillation
APS III: Acute Physiologic Score III
ARB: angiotensin-receptor blocker
ARNI: Angiotensin receptor-neprilysin inhibitor
CI: Confidence interval
CRRT: Continuous renal replacement therapy
DHB CCB: Dihydropyridine calcium channel blocker
HR: Hazard ratio
IQR: Interquartile range
ICU: Intensive care unit
LODS: Logistic Organ Dysfunction Score
MIMIC: Medical Information Mart for Intensive Care
MRA: Mineralocorticoid receptor antagonist
Non-DHB: Non- dihydropyridine
OASIS: The Oxford Acute Severity of Illness Score
PPI: Proton pump inhibitors
SAPS II: Simplified Acute Physiology Score II
SIRS: Systemic Inflammatory Response Syndrome
SOFA: Sequential Organ Failure Assessment

## Data statement

The datasets presented in the current study are available in the MIMIC-IV database (https://physionet.org/content/mimiciv/2.2/).
