# Supplementary material for: Joint effect of atrial fibrillation and obesity on mortality in critically ill patients
Source: Diabetol Metab Syndr. 2024 Jul 18;16:169. doi: 10.1186/s13098-024-01407-8 (PMC11256565; doi:10.1186/s13098-024-01407-8)
Supplement: Supplementary file 1 — Supplementary Material 1 [file 13098_2024_1407_MOESM1_ESM.docx]

**Supplemental Material**

**Figures and figure legends**

**Supplemental Figure 1. Kaplan-Meier curves of 1-year mortality stratified by detailed obesity status in all patients.**


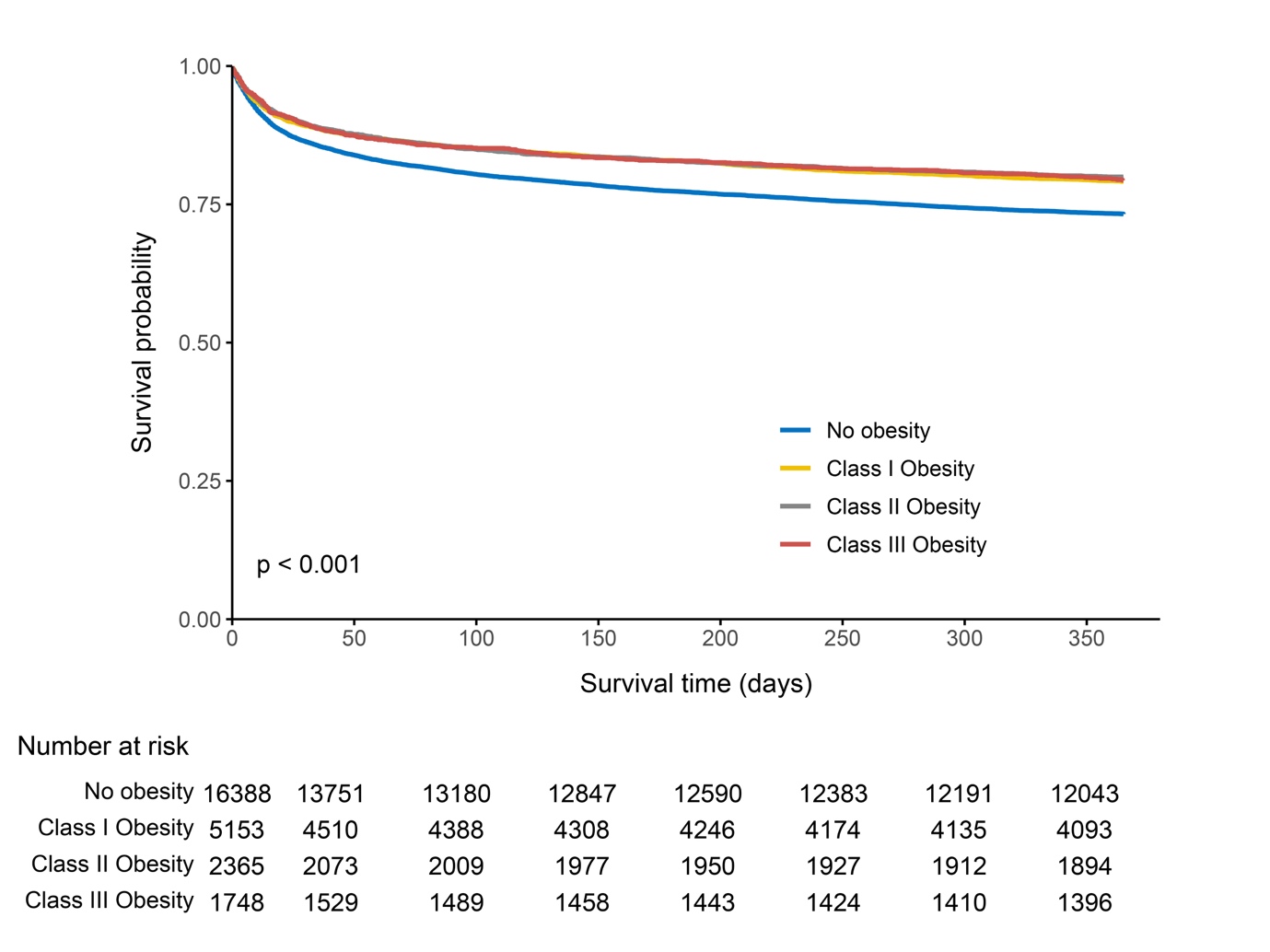


**Supplemental Figure 2. Kaplan-Meier curves of 1-year mortality stratified by detailed obesity status in obese patients.**


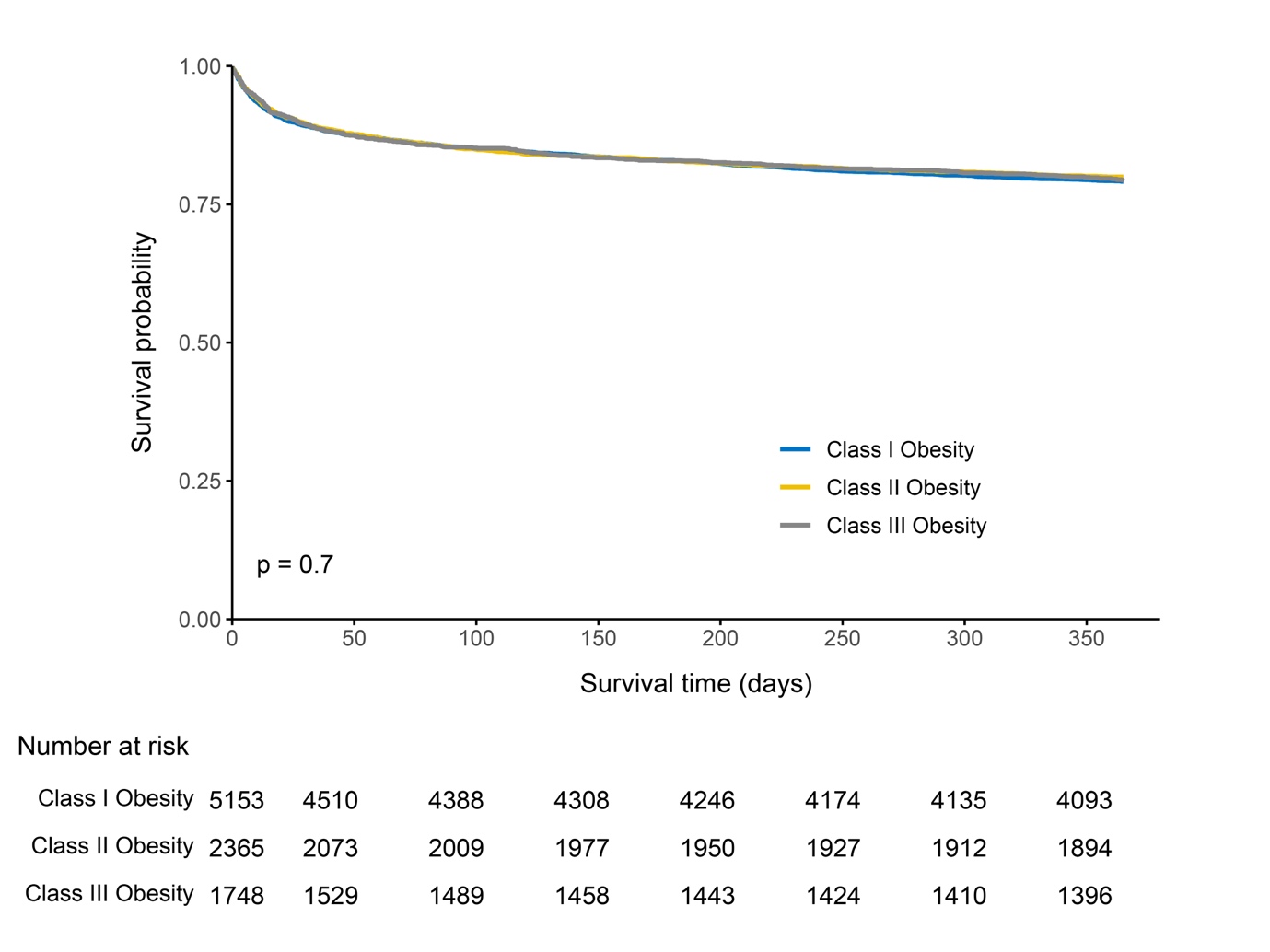


**Supplemental Figure 3. Kaplan-Meier curves of 1-year mortality stratified by AF status according to history of AF in all patients.**


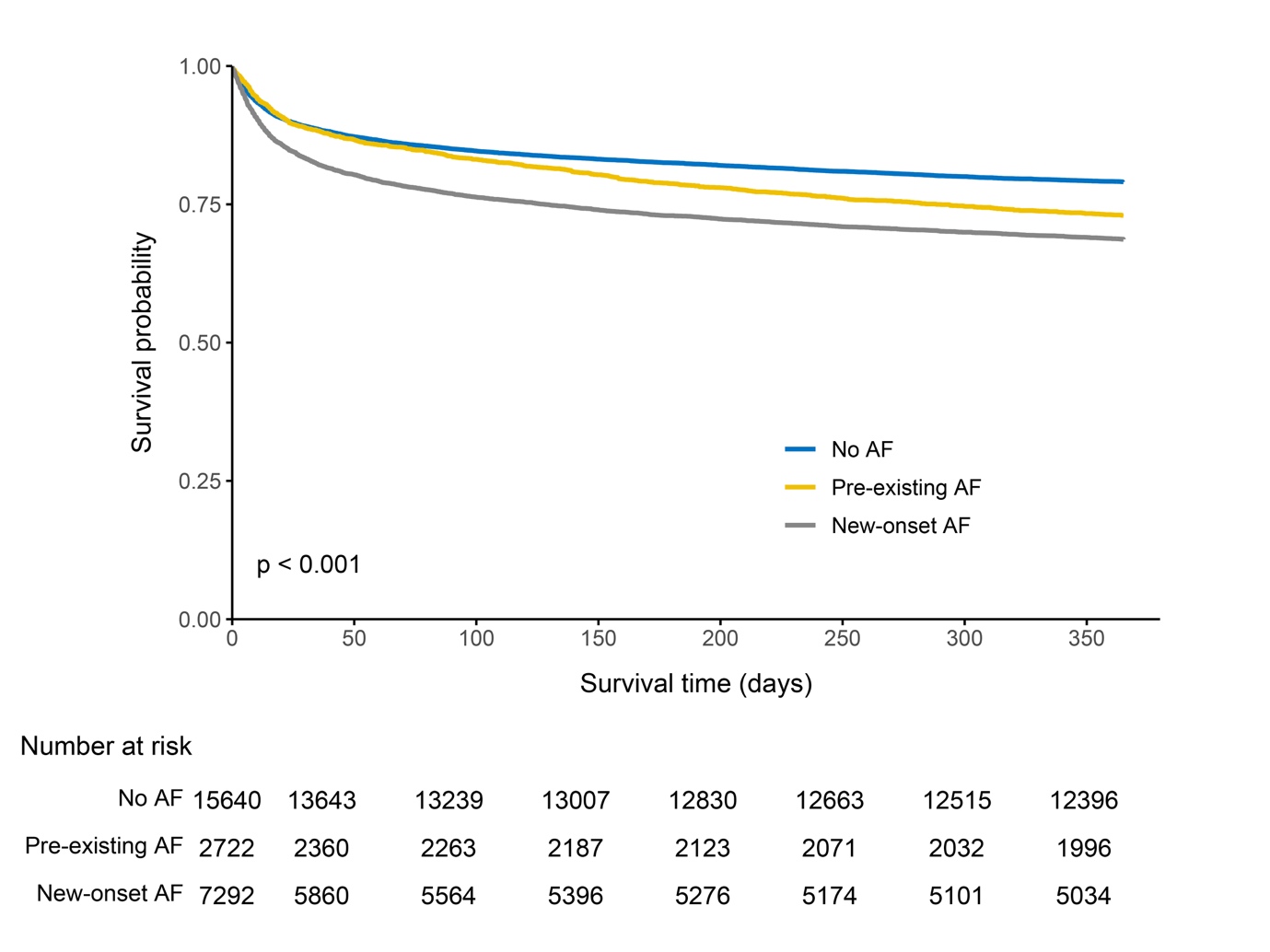


**Supplemental Figure 4. Kaplan-Meier curves of 1-year mortality stratified by AF status according to history of AF in AF patients.**


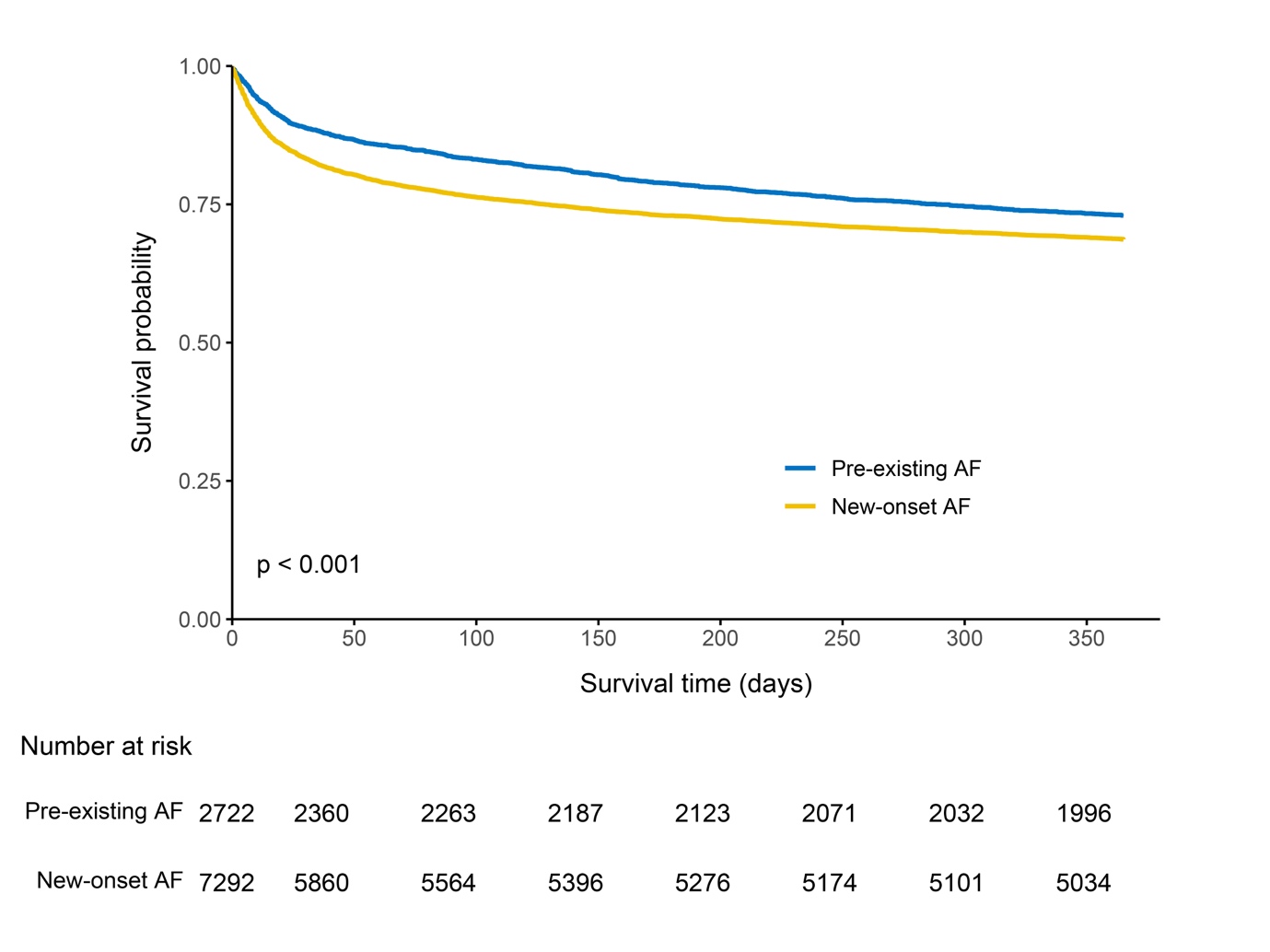


**Supplemental Figure 5. Kaplan-Meier curves of 1-year mortality stratified by AF status according to duration of AF in AF patients.**


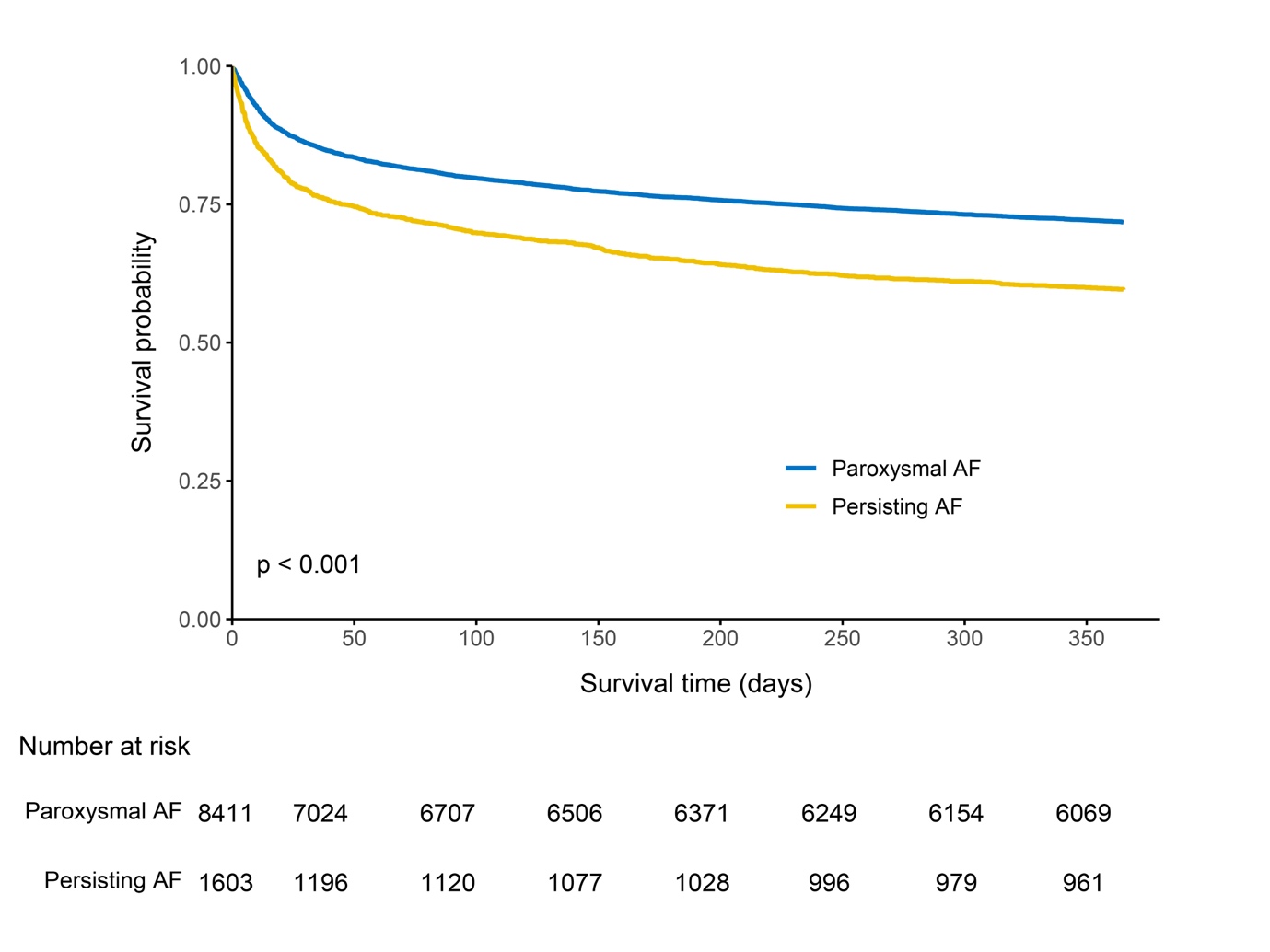


**Supplemental Table 1. Treatment information and comorbidities in AF patients stratified by AF status.**

| **Variables** | **Category by AF history** | | | **Category by AF duration** | | |
| --- | --- | --- | --- | --- | --- | --- |
|  | **Pre-existing AF**  **(n = 2722)** | **New-onset AF**  **(n = 7292)** | **p-value** | **Paroxysmal AF**  **(n = 8411)** | **Persistent AF**  **(n = 1603)** | **p-value** |
| **AF-related Comorbidities** |  |  |  |  |  |  |
| Hypertension, n (%) | 1146 (42.10%) | 3437 (47.13%) | <0.001 | 3989 (47.43%) | 594 (37.06%) | <0.001 |
| Coronary artery disease, n (%) | 1378 (50.62%) | 3689 (50.59%) | 0.975 | 4273 (50.80%) | 794 (49.53%) | 0.351 |
| Myocardial infarction, n (%) | 684 (25.13%) | 1740 (23.86%) | 0.188 | 2087 (24.81%) | 337 (21.02%) | 0.001 |
| Cardiomyopathy, n (%) | 175 (6.43%) | 374 (5.13%) | 0.011 | 391 (4.65%) | 158 (9.86%) | <0.001 |
| Heart failure, n (%) | 1481 (54.41%) | 2770 (37.99%) | <0.001 | 3268 (38.85%) | 983 (61.32%) | <0.001 |
| Sleep apnea, n (%) | 345 (12.67%) | 773 (10.60%) | 0.003 | 924 (10.99%) | 194 (12.10%) | 0.193 |
| Peripheral vascular disease, n (%) | 490 (18.00%) | 1204 (16.51%) | 0.077 | 1404 (16.69%) | 290 (18.09%) | 0.171 |
| Cerebrovascular disease, n (%) | 416 (15.28%) | 1166 (15.99%) | 0.388 | 1306 (15.53%) | 276 (17.22%) | 0.089 |
| Chronic pulmonary disease, n (%) | 895 (32.88%) | 2063 (28.29%) | <0.001 | 2477 (29.45%) | 481 (30.01%) | 0.654 |
| Diabetes, n (%) | 979 (35.97%) | 2346 (32.17%) | <0.001 | 2819 (33.52%) | 506 (31.57%) | 0.129 |
| **AF-related Medications** |  |  |  |  |  |  |
| Oral anticoagulants, n (%) | 1657 (60.87%) | 3328 (45.64%) | <0.001 | 3961 (47.09%) | 1024 (63.88%) | <0.001 |
| Beta-blockers, n (%) | 2316 (85.08%) | 6132 (84.09%) | 0.224 | 7124 (84.70%) | 1324 (82.60%) | 0.034 |
| Non-DHP CCB, n (%) | 679 (24.94%) | 1374 (18.84%) | <0.001 | 1687 (20.06%) | 366 (22.83%) | 0.012 |
| Class IC Antiarrhythmic drugs, n (%) | 42 (1.54%) | 40 (0.55%) | <0.001 | 70 (0.83%) | 12 (0.75%) | 0.733 |
| Class III Antiarrhythmic drugs, n (%) | 1019 (37.44%) | 2951 (40.47%) | 0.006 | 3567 (42.41%) | 403 (25.14%) | <0.001 |
| Digoxin, n (%) | 437 (16.05%) | 707 (9.70%) | <0.001 | 800 (9.51%) | 344 (21.46%) | <0.001 |

AF: atrial fibrillation; Non-DHB: non- dihydropyridine.

**Supplemental Table 2. Outcomes stratified by obesity and AF status.**

| **Outcomes** | **Overall population**  **(n = 25654)** |  | **AF** | |  | **No AF** | |  | **p-value*** |
| --- | --- | --- | --- | --- | --- | --- | --- | --- | --- |
|  |  |  | **Obesity**  **(n = 3735)** | **No obesity**  **(n = 6279)** |  | **Obesity**  **(n = 5531)** | **No obesity**  **(n = 10109)** |  |  |
| ICU mortality | 1983 (7.7%) |  | 317 (8.5%) | 635 (10.1%) |  | 351 (6.4%) | 680 (6.7%) |  | <0.001 |
| In-Hospital mortality | 2688 (10.5%) |  | 423 (11.3%) | 866 (13.8%) |  | 455 (8.2%) | 944 (9.3%) |  | <0.001 |
| 6-month mortality | 5289 (20.6%) |  | 752 (20.1%) | 1799 (28.7%) |  | 832 (15.0%) | 1906 (18.9%) |  | <0.001 |
| 1-year mortality | 6293 (24.5%) |  | 925 (24.8%) | 2094 (33.4%) |  | 985 (17.8%) | 2289 (22.6%) |  | <0.001 |

AF: atrial fibrillation; ICU: intensive care unit.

*Comparisons among the four subgroups.
